# Bumblebee Diversity in Different Habitat Types and Along an Altitudinal Gradient at a Forest–Grassland Ecotone in the East Range of the Qinghai–Tibet Plateau

**DOI:** 10.3390/insects17010049

**Published:** 2025-12-30

**Authors:** Xunlu Xu, Lei Bai, Zhaolong Wang, Jianping He, Yalin Zhang, Xiushan Li

**Affiliations:** 1 Key Laboratory of Southwest Wildlife Resources Conservation of the Ministry of Education, College of Life Science, China West Normal University, Nanchong 637009, China; xxlscsh@163.com (X.X.); bai1358939657@163.com (L.B.); 2 The Station of Qingyang Forest Pest Quarantine and Control, Qingyang 745099, China; 3 Center of Bailong River Forestry Protection of Gansu Province, Lanzhou 730050, China; 4 Key Laboratory of Plant Protection Resources and Pest Management of the Ministry of Education, Entomological Museum, College of Plant Protection, Northwest A&F University, Yangling 712100, China; yalinzh@nwsuaf.edu.cn

**Keywords:** bumblebee, community, overgrazing, vertical distribution, grassland management

## Abstract

Bumblebees are the most important pollinators in alpine areas. Their survival is vulnerable to anthropogenic disturbance. To comprehend the variation in bumblebee diversity in different habitat types and altitudinal gradient, this study carried out transect counting in three distinct habitat types and altitudinal gradient in the source area of the Bailong River between June and September from 2021 to 2022. The result indicates (1) high species richness in bumblebees, as 27 species and 1106 individuals from 9 subgenera were collected. It indicates that the Forest–Grassland ecotone has rich bumblebee diversity. Edge effective and suitable habitats bred rich bumblebees. (2) Species richness and abundance differed between three habitat types: alpine meadows had the highest; shrubs on the forest edge had the second highest; and grasslands had the lowest. (3) The distribution pattern in the altitude of bumblebees shows two peaks. The species richness and abundance of bumblebee are highest in the ranges of 3500–3700 m and 2900–3100 m, while 3100–3500 m is lower than the other altitude ranges, presumably because this range is just a large grassland with overgrazing. (4) The results of redundancy analysis indicate that environmental factors significantly affect the diversity of bumblebees. (5) The conservation measures should mainly focus on alpine meadows, as they are the most important habitat of the bumblebee. Reducing overgrazing in large-scale grasslands is beneficial to bumblebees as well as wildflower plants.

## Simple Summary

This study investigates bumblebee diversity in the headwater region of the Bailong River, China, focusing on the effects of habitat types, altitude, and human disturbance. To comprehend the variation in bumblebee diversity in different habitat types and along an altitudinal gradient, this study carried out transect counting in three distinct habitat types and along an altitudinal gradient in the source area of the Bailong River. The result indicates (1) high species richness of bumblebees in headwater region of Bailong River. It indicates that the Forest–Grassland ecotone has rich bumblebee diversity. Edge effects and suitable habitats supported high bumblebee richness. (2) Species richness and abundance differed between three habitat types: alpine meadows had the highest; shrubs on the forest edge had the second highest; and grasslands had the lowest. (3) The distribution pattern in the altitude of bumblebees shows two peaks. The species richness and abundance of bumblebee are highest in the ranges of 3500–3700 m and 2900–3100 m, while 3100–3500 m is lower than the other altitude ranges, presumably because this range is just a large grassland with overgrazing. (4) The results of redundancy analysis indicate that environmental factors significantly affect the diversity of bumblebees. (5) The conservation measures should mainly focus on alpine meadows, as they are the most important habitat of the bumblebee. Reducing overgrazing in large-scale grassland is beneficial to bumblebees, as well as wildflower plants.

## 1. Introduction

Bumblebees are the most important pollinating insects in the alpine region. They play an important role in the natural ecosystem owing to their distinct flower-visiting habits and adaptation to frigid environments. The reproduction of wild plants in alpine regions mainly depends on bumblebees’ pollination to maintain sustainable survival and ecosystem stability [1]. Some species that are suitable for artificial breeding have significant economic value in pollinating vegetables and fruits in greenhouses [2,3]. Pollination by bumblebees can significantly improve the yield and quality of tomatoes [4].

Bumblebees are widely distributed in the temperate and cold temperate regions of the Northern Hemisphere [5,6,7]. Their habitat types are mainly large grassland and alpine meadows in highlands. In recent years, the degeneration of bumblebee foraging habitat due to the intertwined effects of agricultural intensification and climate change has led to a downward trend in bumblebee diversity levels [8,9]. Environmental factors such as altitude, temperature, and humidity in pollinator habitats are important influences on the distribution of flower-visiting insects [10]. In natural environments, the structure and diversity of flower-visiting insect communities are subject to the superimposed effects of several environmental factors [11].

Loss of biodiversity alters ecosystem functioning and stability [12,13,14], leading to reduced productivity, reduced capacity for pollination services, and imbalances in nutrient cycling throughout the ecosystem [12,13,15,16,17]. In North America, due to intensive agriculture and the influence of foreign pathogens, the diversity of bumblebees is constantly decreasing, and about one-third of local bumblebee species are considered vulnerable or endangered by the International Union for Conservation of Nature [18]. In particular, most subgenera in *Bombus* and *Psithyrus* have been recognized as endangered and protected species [19,20,21]. In the UK, the sharp decline in non-arable land may lead to the destruction of bumblebee habitats and a significant decrease in bumblebee diversity levels. Of the 16 species of bumblebees, 6 showed a significant decrease, 4 were likely to decrease, and only 6 remained stable or increased in number [22].

Species diversity along an altitudinal gradient is a major focus of spatial ecology and biogeography studies [23]. In Japan, bumblebee diversity was explained by surveying altitude or other factors (e.g., season, survey area, and flower abundance), and a model that included only altitude was found to best explain bumblebee diversity [24].

Bumblebees belong to the Order Hymenoptera, family Apidae, and genus *Bombus*, with approximately 15 subgenera and 250 species worldwide [25,26].

China is a country with rich biodiversity. There are 125 species of bumblebee in China. The Qinghai–Tibet Plateau is a global hotspot of biodiversity [27,28]. Its northeastern edge is a Forest–Grassland ecotone and the richest area of bumble bees in China, where a large number of endemic species of the Tibetan Plateau have been discovered [26,29]. Our survey area encompasses the source region of the Bailong River, situated at the northeastern periphery of the Qinghai–Tibet Plateau. This region’s vegetation is composed of old-growth forest, grasslands, agricultural lands, There are 27 species of bumble bees in this region [27]. Historically, this region experienced heavy wood cutting and persistent overgrazing and, currently, tourism development is growing quickly. All of these human disturbances result in the degradation of the natural ecosystem. To know the impact of habitat change and human disturbance on the bumblebee community, we selected this region as the research area.

Our research hypothesis was that (1) the change in habitat type has an obvious influence on the bumblebee community; (2) we expected alpine meadows to host the highest diversity due to floral abundance; (3) local overgrazing impacts the bumblebee decline; and (4) the distribution patterns in the altitudinal gradient are negatively related to altitude.

Our research, drawing from actual survey data, explores the diversity of bumblebees in this region with the objectives of: (1) elucidating the current status of bumblebee diversity; (2) examining how bumblebee species diversity varies across habitat types and altitude gradients; and (3) assessing the types and intensity of human-induced disturbances affecting bumblebee diversity. The goal is to provide a base for the conservation and utilization of bumblebee species diversity.

## 2. Materials and Methods

### 2.1. Overview of the Study Area

**Figure 1:**
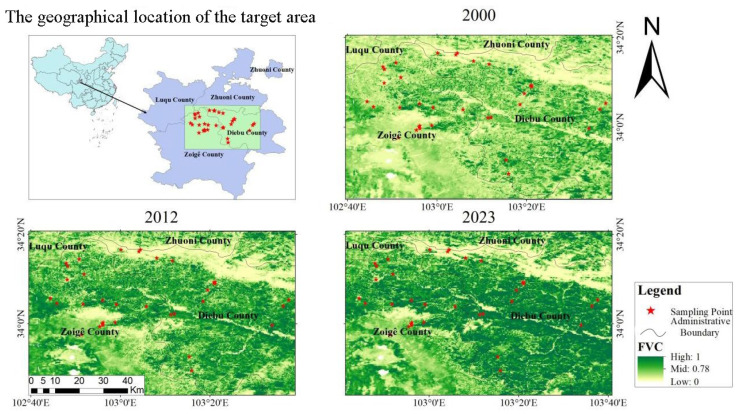
Bumblebee transect distribution and vegetation change in the headwaters of the Bailong River. Vegetation coverage in the whole area: 2000: 73.86%, 2012: 78.93%, 2023: 81.53%. Colors: green: forest and grassland, yellow: agricultural land and village.

The survey area is located in Diebu and Ruoergai (Zoige) County. Diebu County is located in the south of Gannan Tibetan Autonomous Prefecture, southwestern Gansu Province of China. Ruoergai County is located in Aba Prefecture, northwestern Sichuan Province. This area is located at 33°49′–34°08′ N and 102°44′–103°337′ E. The terrain includes the high mountain canyon area of the Dieshan Mountains in the most western part of the Qinling Mountains and the Minshan Mountains in the northwest of Sichuan. The survey locations range in altitude from 2216.4 m to 4166.5 m. It belongs to the transitional climate type of high-altitude cold climate on the eastern edge of the Qinghai–Tibet Plateau. This area consists of old-growth forests, large-scale grasslands, and alpine meadows. Historically, this area experienced long-term severe wood cutting and overgrazing. Since 1998, logging has been halted, and forest restoration began. The vegetation cover canopy increased from 73.86% in 2000 to 78.93% in 2012 and continued to 81.53% in 2023. See Figure 1.

**Figure 2:**
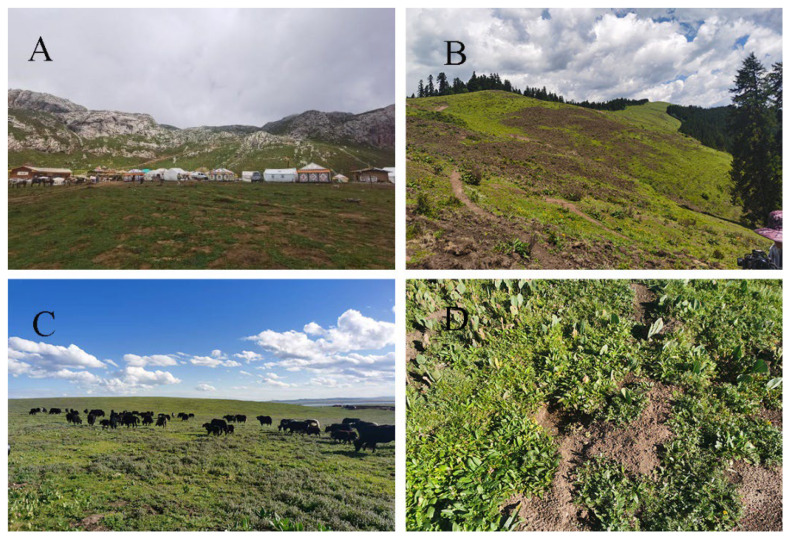
Threats to grassland in the source region of the Bailong River: (**A**) tourism development; (**B**) wild pig destroy grassland; (**C**) livestock overgrazing; (**D**) Grassland rodent infestation.

Due to population growth and economic development, heavy human disturbance has persisted. Currently, tourism is quickly developing and livestock overgrazing is the main threat to grassland. Additionally, wild pigs seriously destroy the grassland. Figure 2.

### 2.2. Transect Selection and Survey Methods

**Figure 3:**
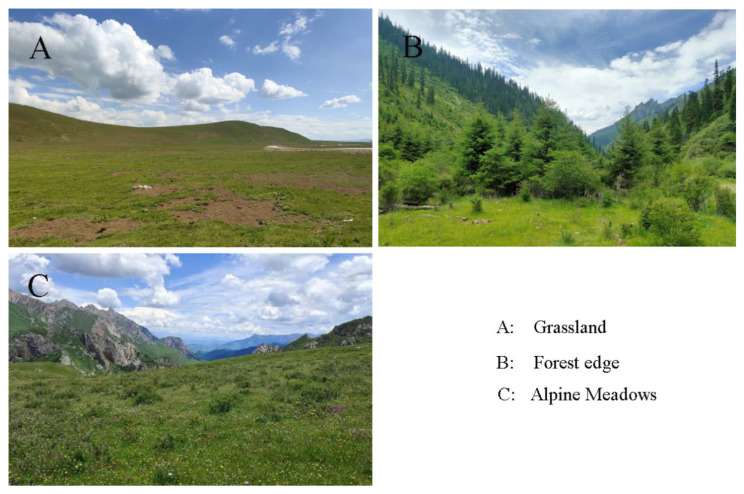
Tree types of habitats.

The field survey time was between June–September in 2021–2022 which is the peak period of bumblebee flower-visiting activity. Grasslands, alpine meadows, and forest edge (including shrub and grass) (Figure 3) in Diebu and Ruoergai Counties were selected as the main habitat types.

Based on local topographic factors, stratified random sampling was applied to set up transects covering all three habitats and every 200 m altitude difference in the survey area, with a total of 33 transects, each of which was 5 m in width and 200 m in length. Habitat types and intensity of disturbance follow the Technical Provisions for Survey and Assessment of Insect Diversity in Counties issued by the Ministry of Ecology and Environment (https://www.mee.gov.cn/gkml/hbb/bgg/201801/t20180108_429275.htm) (1 January 2020) Transect counting used the standard bee-walk survey methodology [30]. Grazing disturbance intensity is determined by livestock density in local pastures (exceeding 2.9 sheep unit/ha, 1 cow = 4 sheep units, 1 horse = 5 sheep units). For species identifications, we invited a bumblebee taxonomy expert to assist us. The distribution of transects is shown in Figure 2.

### 2.3. Bumblebee Diversity Analysis

Four species diversity indices were selected to compare the alpha diversity of bumblebee communities in different habitat types and gradients of elevations and interference: the Shannon–Wiener diversity index (*H*’), the Pielou evenness index (*E*), the Simpson dominance index (*D*), and the Margalef richness index (*R*). The formula is as follows:

H’ = −∑P_i_ (lnP_i_)

E = H’/lnS

D = 1 − ∑P_i_^2^

R = (S − 1)/lnN

where P_i_ is the ratio of the number of individuals of the ith species to the total number of individuals, S is the number of all species, and N is the total number of individuals [31].

### 2.4. Analysis of Bumblebee Communities

The beta diversity of bumblebee community composition was analyzed by non-metric Multi-Dimensional Scaling (NMDS) ranking method and non-parametric Analysis of Similarities (ANOSIM). The correlation between different environmental factors and the bumblebee community was analyzed by redundancy analysis (RDA). The magnitude of the correlation between bumblebee community diversity and environmental factors depends on the cosine of the diversity line and the environmental factor line. In addition to these traditional indices, we expressed alpha diversity using the Hill numbers framework (also termed “effective number of species”) [32], which places richness, Shannon, and Simpson diversity on a common, linear scale that is easier to interpret and compare among habitats and elevation bands. Hill numbers convert each index into the number of equally abundant species that would yield the observed value, allowing rare, common, and dominant species to be contrasted within a single unified system. In this framework, order q = 0 corresponds to species richness (sensitive only to presence–absence), q = 1 to the exponential of Shannon entropy (reflecting the effective number of rare species), and q = 2 to the inverse of Simpson concentration (reflecting the effective number of dominant species). This approach has become standard in biodiversity comparisons because it avoids many of the non-intuitive properties and incompatibilities of the raw indices.

### 2.5. Statistical Analysis of Data

The data were organized using Microsoft Excel 2019, and statistics were performed using SPSS 26.0. Using ArcGIS software (10.2), bioclimatic and topographic factors of each sample site were extracted from the data maps based on the latitude and longitude of the sample sites, where the climate data was obtained from WorldClim version 2.1 [33], the topographic base map of climate data was from worldclim (http://www.earthenv.org/), and the base map of terrain data was from EarthEnv (http://www.earthenv.org/). Diversity index calculation, NMDS, and ANOSIM analyses were carried out using the “Vegan” package of R software(R-4.5.2) [34]; environmental factors were used as explanatory variables, and number of individuals, number of species, and biodiversity index were used as response variables using Canoco5 software. Numbers were log-transformed and the biodiversity index was centered for RDA redundancy analysis. R4.2.2, Canoco5, and Origin software (origin 2022) were applied for mapping. The Hill number was estimated using the “iNEXT” package of the R software for sparse extrapolation based on the relationship between the number of bumblebee individuals and the number of species in the actual collection [35].

## 3. Results and Analysis

### 3.1. Species Composition of Bumblebee Community

**Figure 4:**
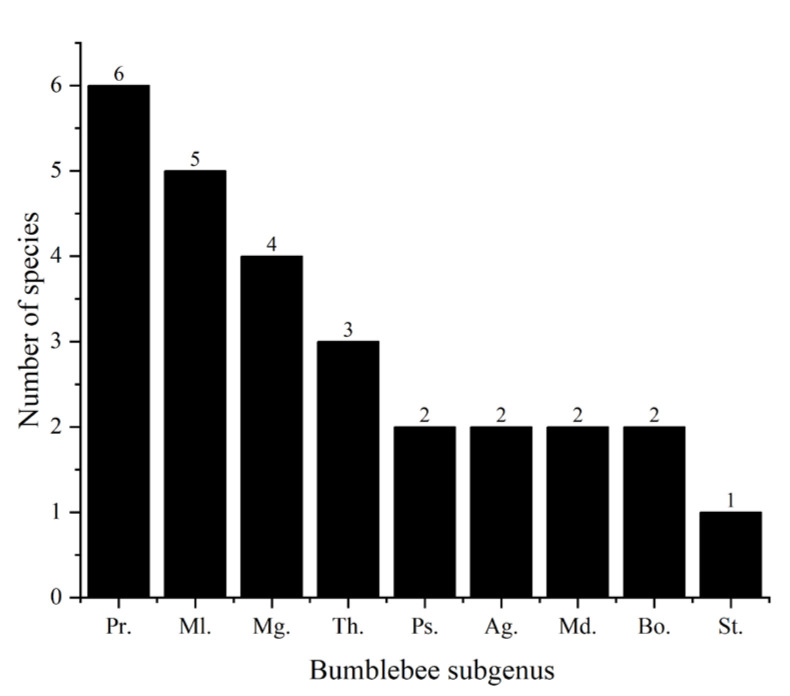
Species richness per bumblebee subgenus in the headwaters of the Bailong River: *Pyrobombus* (Pr.), *Melanobombus* (Ml.), *Megabombus* (Mg.), *Thoracobombus* (Th.), *Psithyrus* (Ps.), *Alpigenobombus* (Ag.), *Mendacibombus* (Md.), *Bombus* s. str. (Bo.), and *Subterraneobombus* (St.).

A total of 1106 individuals of the genus *Bombus* were collected from 33 transects, belonging to 27 species and 9 subgenera (Figure 4). See Appendix A.

### 3.2. Bumblebee Diversity

#### 3.2.1. Bumblebee Diversity in Different Habitats

The number of species and individuals of the genus *Bombus* varied considerably between habitat types. Alpine meadows had the highest species richness and abundance, accounting for 55.33% of the total collection. Forest edge had the second-highest species richness and abundance, accounting for 32.46% of the total collection. Grasslands had the lowest species richness and abundance, accounting for 12.21% of the total collection (Table 1).

**Figure 5:**
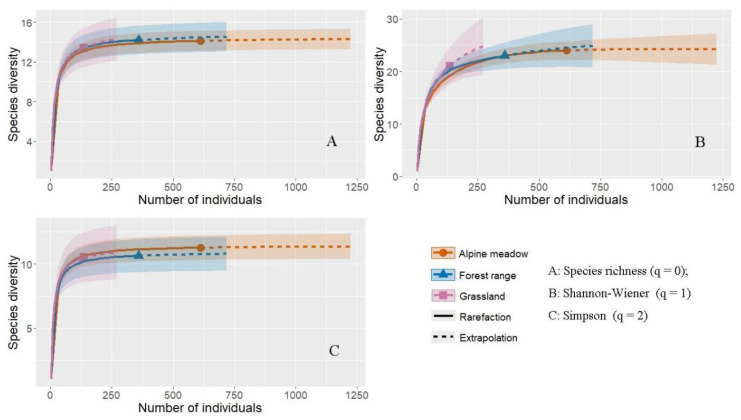
Bumblebee diversity in different habitats in the headwaters of Bailong River. (**A**) Species richness (q = 0); (**B**) the exponential of Shannon–Wiener (exp H’) (q = 1); (**C**) The inverse of Simpson’s concentration index (1/D) (q = 2).

The rarefaction and extrapolation curves based on Hill numbers revealed differences in bumblebee community diversity among the three habitat types. Species richness differed significantly among the three habitats (Kruskal–Wallis test: H = 12.7, *p* = 0.002). Post hoc Dunn’s tests revealed that alpine meadows supported significantly higher richness than both grasslands (*p* = 0.005) and forest edges (*p* = 0.018), followed by forest-edge habitats. Although the highest number of individuals was found within alpine meadows, it had the lowest extrapolated Shannon–Wiener diversity and species richness. This phenomenon may arise from hyper-aggregation of dominant bumblebee species. Simpson diversity was highest in alpine meadows and lowest in forest edges (Figure 5).

**Figure 6:**
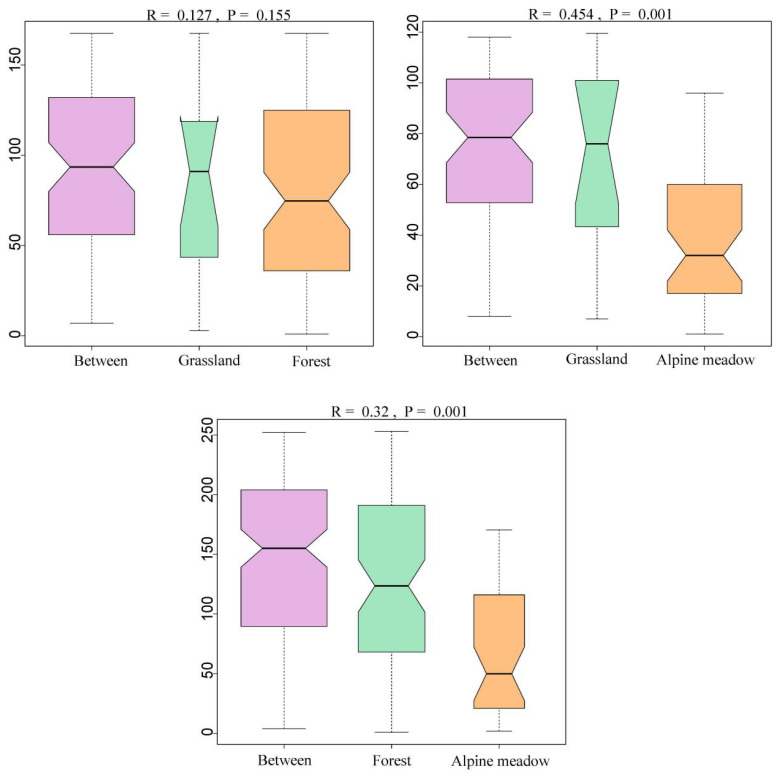
Analysis of the similarity of bumblebee colonies in different habitats in the headwaters of Bailong River. Habitat types can be divided into two categories: Alpine meadows and Grassland/Forest. The values of intergroup differences were higher than the intragroup values.

According to the results of ANOSIM analysis (Figure 6), the values of intergroup differences between grasslands and forest range were higher than the intragroup values between the two habitat types (R = 0.127), but there was no significant difference (*p* > 0.05). The values of intergroup differences between grassland and alpine meadows, and between forest range and alpine meadows, were higher than the intragroup values between them (R = 0.454, R = 0.32), which indicates that bumblebee communities in alpine meadow composition differed significantly (*p* < 0.01) from grassland and forest range habitats.

**Figure 7:**
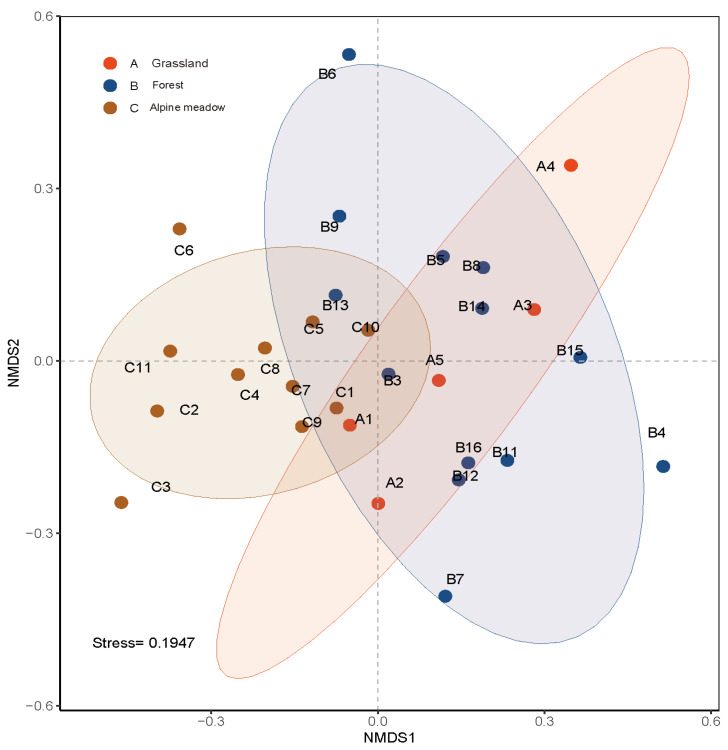
Analysis of the similarity of bumblebee colonies in different habitats in the headwaters of Bailong River. The composition of bumblebee communities in different habitat types can be divided into two categories: alpine meadows alone and grasslands and forest range grouped together.

The NMDS stress function value for bumblebee communities among different habitats was 0.2 (Figure 7). These results could be shown in the NMDS two-dimensional point map, but it is difficult to represent the differences among bumblebee community structures by a single principal axis only. The bumblebee communities in the three habitat types show an overall relatively aggregated distribution, with intersecting parts in each habitat. However, there exist some sample points that are discrete compared to other sample points in the same habitat. In general, the composition of bumblebee communities in different habitat types can be divided into two categories: Alpine meadows and Grassland/Forest, which are relatively separated on the NMDS ordination diagram.

#### 3.2.2. Distribution Pattern on Altitude of Bumblebees

The species richness and abundance of bumblebees varied significantly among different altitudes, showing bimodal distribution. Theyare highest in the range from 3500 to 3700 m, followed by 2900–3100 m. And lowest in the range from 2500 to 2700 m, followed by 3300–3500 m. While the rest of the altitude range was moderate (Table 2).

**Figure 8:**
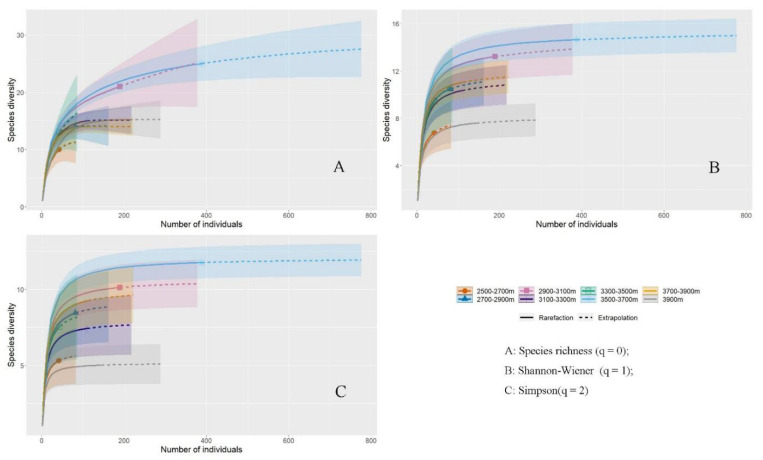
Bumblebee diversity at different altitudes in the headwaters of Bailong River. (**A**) Species richness (q = 0); (**B**) the 252 exponential of Shannon–Wiener (exp H’) (q = 1); (**C**) The inverse of Simpson’s concentration index 252 (1/D) (q = 2).

Sparse extrapolation of the relationship between species number and abundance across the altitudinal gradient shows that the Shannon–Wiener diversity, Simpson diversity, and species richness are highest in the middle to high altitude range (3500–3700 m); Shannon–Wiener diversity is lowest in the low altitude range (2500–2700 m); and Simpson diversity and species richness are lowest in the high altitude range (3900 m and above). Areas above 3900 m have the lowest Simpson diversity and species richness (Figure 8).

**Figure 9:**
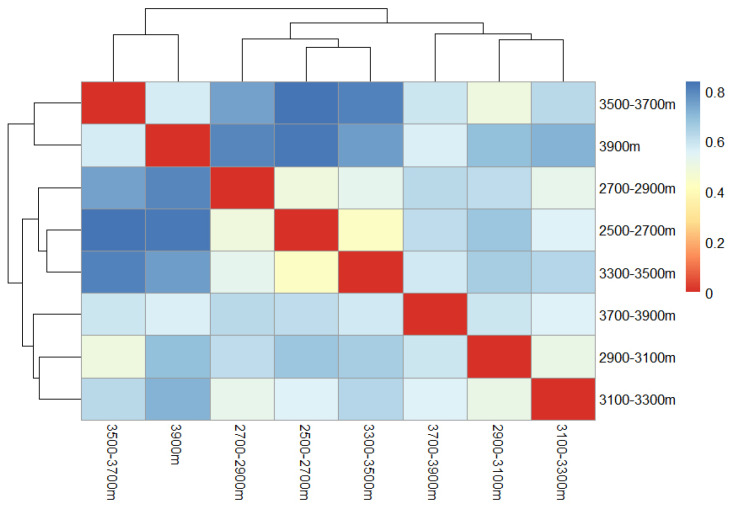
Similar cluster of bumblebees at different altitudes in the headwaters of Bailong River.

The coefficients of dissimilarity between bumblebee communities in the ranges of 2500–2700 m, 3500–3700 m, and above 3900 m at low altitudes, as well as in the ranges of 2700–2900 m and above 3900 m, and in the ranges of 3300–3500 m and 3500–3700 m, were all above 0.75, which indicates that bumblebee communities in these altitude ranges were very dissimilar to each other. The coefficients of dissimilarity between bumblebee communities in the other altitude ranges were between 0.5 and 0.75. Bumblebee communities in the 2500–2700 m and 3300–3500 m altitude ranges were the most similar in terms of vertical distribution, and clustered together with a coefficient of dissimilarity of 0.4285; bumblebee communities in the 3100–3300 m and 2900–3100 m altitude ranges clustered together at a coefficient of dissimilarity of 0.5167; and bumblebee communities in the 3500–3700 m and 3900 m altitude ranges were the most similar in terms of vertical distribution at a coefficient of dissimilarity of 0.5167. The coefficient of dissimilarity was 0.5751, clustered into one group (Figure 9).

#### 3.2.3. Effects of Environmental Factors on Bumblebee Diversity

**Figure 10:**
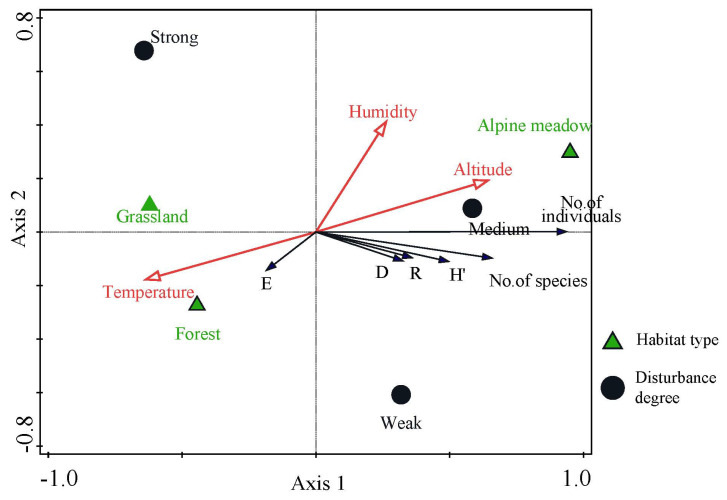
Redundancy analysis ordination diagram of community diversity of bumblebees and environmental factors in the headwaters of Bailong River. Note: H’, Shannon index; E, Pielou index; D, Simpson index; R, Margalef index. Vectors (arrows) represent environmental variables and are scaled to reflect their relative strength of correlation with the community composition. Arrow length is proportional to the effect size of each variable in explaining variation in the bumblebee community.

According to the redundancy results, vectors are scaled and the arrow length = effect size. The number of species richness and abundance of bumblebees and the Shannon–Wiener diversity index, Simpson dominance index, and Margalef richness index of the community are all positively correlated with the first principal component, whereas the Pielou evenness index was negatively correlated with the first axis. The multiplicity of bumblebee species is close to the first axis, i.e., it had the highest correlation with the first principal component, followed by the number of bumblebee species and the lowest correlation with the first axis. In the number of bumblebee species, Pielou’s evenness index had the lowest correlation with the first axis. The number of bumblebee species, Shannon–Wiener diversity index, Pielou evenness index, Simpson dominance index, and Margalef richness index were negatively correlated with the second principal component. But the second axis eigenvalues were low, i.e., all response variables did not correlate well with the second axis (Figure 10).

RDA redundancy analysis results indicate elevation and humidity are positively correlated with the first principal component, while temperature is negatively correlated with the first principal component. Elevation and temperature have the highest correlation with the first principal component, indicating that the first axis characterizes the temperature and elevation gradient. Humidity and elevation are positively correlated with the second principal component, and temperature is negatively correlated with the second principal component. Of these, humidity has the highest correlation with the second principal component, and elevation and temperature have lower correlations with the second principal component. The number of bumblebee species and individuals, as well as the Shannon–Wiener diversity index, Simpson’s dominance index, and Margalef’s richness index of the community, are positively correlated with elevation and humidity. The elevation factor is more highly correlated with the humidity factor and negatively correlated with temperature. Pielou’s evenness index is negatively correlated with elevation and humidity and positively correlated with the temperature factor.

In terms of the distribution of habitat types, alpine meadows are located in the first quadrant, grasslands in the second quadrant, and forest range in the third quadrant. The grasslands and forest range are located relatively close to each other, suggesting that there is a high degree of similarity in the diversity of bumblebee communities between the two habitats, and a high degree of difference in the diversity of bumblebee communities with those in the alpine meadow habitat. Different habitats also affect the diversity of bumblebee communities differently. The number of bumblebee species and individuals, as well as the Shannon–Wiener diversity index, Simpson’s dominance index, and Margalef’s richness index of communities within alpine meadows, are higher than those of woodlands and grasslands. Pielou’s evenness index of bumblebee communities indicates that evenness within grasslands and woodlands is higher than that of alpine meadows.

The RDA ordination plot shows that the relative positions of different disturbance levels are more dispersed. Which means both weak and moderate disturbance in the positive half-axis of the first axis and positively correlated with species richness and abundance, while strong disturbance is located in the second quadrant andnegatively correlated with species richness and abundance. The effects on the number of bumblebee species and the Shannon–Wiener diversity index, Pielou evenness index, Simpson dominance index, and Margalef richness index of the community are higher at the level of weak disturbance than at the level of moderate disturbance, while the effects on the number of bumblebee individuals are higher at the level of moderate disturbance compared to weak and strong disturbance.

## 4. Discussion

### 4.1. Bumblebee Species Richness

After the surveys in 2021–2022, a total of 27 species of bumblebees in 9 subgenera were collected in the source area of the Bailong River, accounting for 22.4% of the total number of bumblebee species in China [27]. Species richness is higher than that of the Baishui River Nature Reserve located in the lower part of the Bailong River [36].

### 4.2. Bumblebee Diversity in Three Type of Habitats

This survey reveals obvious differences in the bumblebee community composition between three habitat types: alpine meadows have the highest species richness and abundance, accounting for 55.33% of the total collection; shrub on the forest edge have the second highest species richness and abundance, accounting for 32.46% of the total collection; and grasslands have the lowest species richness and abundance, accounting for 12.21% of the total collection, which likely results from overgrazing. The three habitat types have nearly the same species richness, but there is a substantial difference in abundance. This indicates that when human disturbance (over-grazing) is severe, the abundance decreases at first, and then the number of species decreases after reaching a certain limit.

Alpine meadows are one of the most important ecosystems in the Qinghai–Tibetan Plateau, and have higher plant species diversity, functional richness, and functional dispersion than grassland ecosystems [37]. They consists of shrubs and grass at higher altitudes, 3500 m. livestock not easy arrival there and shrub can defend grass. Therefore overgrazing is less there. The large-scale grasslands are normally used for pasturing livestock; The forest habitats are normally small areas and are grazed less by livestock.

The species richness and community composition of pollinators in different habitats are easily and strongly affected by changes in the composition and structure of vegetation in the habitats [38]. There is a correlation between bumblebee species diversity and nectar plant richness, and when there is a downward trend in bumblebee-preferred plant diversity, bumblebee species richness also decreases significantly [39]. The species composition and diversity levels of bumblebees in the three habitats differ significantly, probably due to differences in the composition of vegetation and the abundance of plant resources in the habitats.

### 4.3. Bumblebee Diversity Across Altitudinal Gradient

Bumblebees would rather live in habitats at a higher altitude. Generally, their species richness and abundance increased with altitude. But the distribution of bumblebees in altitude shows a two-peak pattern. The number of species and abundance of bumblebees is highest in the ranges of 3500–3700 m and 2900–3100 m, while lower between 3100 and 3500 m. This is because from 3100 m to 3500 m it is a large-scale grassland with overgrazing. Heavy human disturbance results in a decline of bumble bee diversity.

The bumblebee communities in the ranges of 2900–3100 m, 3100–3300 m, 3500–3700 m, and 3900 m above sea level were less dissimilar, i.e., the bumblebee communities were more similar between adjacent altitudes, possibly due to the similarity of habitats and the presence of overlapping floristic resources between adjacent altitudes. The highest similarity was found in the ranges of 2500–2700 m and 3300–3500 m, which may be due to the wider flight range of bumblebees [40]. The ability to fly long distances in search of more nectar sources may also be due to the fact that bumblebee colonies in both altitudinal ranges have been subjected to greater anthropogenic disturbances, resulting in lower levels of bumblebee species and abundance within the colonies, and lower diversity indices in both cases, leading to a high degree of inter-colony similarity.

Meanwhile, the diversity level of bumblebee communities within each altitudinal gradient was not consistent. During June to September when bumblebees were active in the source area of the Bailong River, the Shannon–Wiener diversity index, the Pielou evenness index, and the Simpson dominance index of bumblebee communities were the highest in the altitudinal range of 3500–3770 m, whereas the 3300–3500 m altitudinal range of the Margalef richness index was the highest, indicating that the environment was more suitable for bumblebee survival at middle and high altitudes. This difference may be due to the cold climate of high altitudes; bumblebees are better adapted to survive in low-temperature environments than other flower-visiting insects [1]. This results in less competitive pressure on floral resources compared to lower altitude, e.g., the proportion of bumblebees among flower-visiting insects in the sea cactus primrose (*Primula poissonii*) increased with altitude, while the proportion of other pollinators (Lepidoptera) decreased with elevation [41]. It may also be caused by changes in the allocation of plant resources at different altitudes. In order to alleviate the pollen limitation that increases with altitude, the endemic plant species of the Tibetan Plateau, *Trollius ranunculoides*, changes the allocation of resources to increase the pollination rate [42]. This results in higher bumblebee aggregation and community diversity at middle and high elevations.

### 4.4. Influence of Environmental Factors on Bumblebee Communities

Based on actual investigation, the degree of disturbance in the region is divided. It is found that the species richness and abundance of bumblebee communities are higher under weak and moderate disturbance than under excessive disturbance. Excessive human disturbance may damage the original habitat, causing the loss of habitat for bumblebees and a decrease in their diversity. In Hongyuan County, which is close to our survey area, overgrazing significantly reduces the richness of nectar plants, leading to a decrease in the richness of bumblebee species [39]. The lack of nectar plants due to excessive disturbance is also a major factor in the decline of bumblebee diversity. When a decline in bumblebee abundance occurs, it affects pollinator plant diversity, further leading to a decline in bumblebee species diversity.

Through the RDA of bumblebee diversity in relation to temperature, humidity, and altitude, it is observed that the Shannon–Wiener diversity index, Simpson dominance index, Margalef richness index, species richness, and abundance of bumblebee are positively correlated with altitude, increasing with altitude. Conversely, they are negatively correlated with temperature, increasing as temperature decreases. This suggests that bumblebees are sensitive to temperature and have cold resistance, enabling them to better adapt to cold environments. Relevant studies have shown that with global warming, most bumblebee species in southern Europe have migrated to higher altitudes, with an average elevation increase of 300 m [9]. This means bumblebees face a high extinction risk due to climate change, because they live at the mountain top and have no place to migrate [43].

Pielou’s evenness index decreased with increasing altitude. This pattern aligns with thermal adaptation principles such as Bergmann’s rule [44]. Our community composition data show a shift toward the dominance of known larger-bodied bumblebee species (e.g., *Bombus convezus* and *B. supremus*) at higher elevations. These species possess a larger body size, a trait that confers superior heat conservation in colder environments. Consequently, the increased numerical dominance of these cold-adapted, larger-bodied species led to a reduction in overall community evenness at high altitudes. In bivariate regression analyses, the number of bumblebee species and individuals were significantly (*p* < 0.05) correlated with the warmest season mean temperature and the warmest season precipitation, similar to previous studies [45], suggesting that precipitation also affects the geographic distribution of bumblebees to some extent.

The above analysis confirms that three of our four hypotheses align with reality, but the distribution of bumblebees along the elevation gradient contradicts our hypothesis—that species richness and abundance increase with elevation. However, between 3100 and 3500 m, these metrics decline due to overgrazing.

### 4.5. Recommendations for the Conservation of Bumblebee Diversity

This research found that each transect location was subject to varying degrees of human interference. Local Tibetan communities have transformed from a nomadic lifestyle to a settled lifestyle. They enclose private pastures with fences and take turns to intensively graze each pasture while allowing other pastures to rest; this is achieved by dividing them into summer and winter pastures (distance of summer pasture is longer than 15 km from village while winter pasture is less than 15 km). This to some extent reduces the average intensity of grazing, but summer pastures are generally set up in high-altitude mountainous areas, which happen to have the highest diversity of bumblebees. Large-scale grazing may have a certain degree of impact on bumblebee diversity. Concurrently, the local promotion of tourism and the construction of roads and bridges may exert certain impacts on the habitats of bumblebees and the vegetation within their ranges, leading to a reduction in bumblebee species diversity. It is recommended that local strategies for protecting vulnerable bumblebee species be introduced, encompassing areas with high species richness and the most dominant habitat coverage within the current scope. For example, designating the 3500–3700 m elevation belt as priority conservation zones.Limiting the over-grazing degree in large-scale grassland.

## Figures and Tables

**Table 1:** Composition of bumblebees in different habitats of the headwaters area of the Bailong River.

Habitat Types	Number of Species	Abundance
Grassland	21	135 ± 12
Alpine meadow	24	612 ± 45
Forest edge	23	359 ± 28

**Table 2:** Composition of bumblebees in different habitats of the Bailong River headwaters area.

Elevation Gradient	No. of Species	No. of Individuals
2500–2700 m	10	41
2700–2900 m	14	81
2900–3100 m	20	189
3100–3300 m	15	109
3300–3500 m	12	43
3500–3700 m	24	388
3700–3900 m	14	111
Above 3900 m	15	144

## Data Availability

The original contributions presented in this study are included in the article. Further inquiries can be directed to the corresponding author.

## Appendix A. Information of Transects and Transect Counting Results

**Number**	**Place**	**Elevation (m)**	**Latitude**	**Longitude**	**Number of Species**	**Number of Individuals**
1	Amitang village	3027.7	N: 34.086911°	E: 102.934802°	11	34
2	Amitang	2877.6	N: 34.072425°	E: 102.985878°	1	3
3	Top of Amitang	3523.1	N: 34.072632°	E: 102.861723°	12	60
4	Across the Bailongjiang River	2932.1	N: 34.183102°	E: 102.865450°	6	33
5	Dala Valley	3077.2	N: 33.826289°	E: 103.265317°	7	26
6	Dangduo Valley	3183.8	N: 34.240597°	E: 102.847530°	4	9
7	Top of Dieshan	4166.5	N: 34.276574°	E: 103.075590°	12	85
8	Dieshan ranch	4008	N: 34.269370°	E: 103.069908°	8	59
9	Hongxin ranch	3228.1	N: 34.074521°	E: 102.766655°	11	51
10	Hongxin village	3175.5	N: 34.095120°	E: 102.739920°	5	15
11	Hutoushan	2727.6	N: 34.034215°	E: 103.188822°	10	52
12	Hutoushan ranch	2942.7	N: 34.036399°	E: 103.201568°	8	24
13	Ranger station	2690.4	N: 33.877585°	E: 103.256695°	4	15
15	Huahu	3501.3	N: 33.960116°	E: 102.856405°	9	17
16	Jianni valley	2216.4	N: 33.994348°	E: 103.565417°	1	3
17	Top of Jianni valley	2748.8	N: 34.088883°	E: 103.627663°	5	26
18	middle of Jianni valley	2550	N: 34.066339°	E: 103.606333°	1	2
19	Jiangza	3048.3	N: 34.163896°	E: 102.808650°	7	16
20	Top of Jiangza	3662.6	N: 34.224938°	E: 102.799085°	10	69
21	Middle of Jiangza	3551.8	N: 34.214667°	E: 102.803680°	16	61
22	Reer	2556.1	N: 34.064755°	E: 103.097080°	6	19
23	Stone Mountain	3284.2	N: 34.005609°	E: 102.980660°	10	34
24	Teniza	3557.6	N: 34.004781°	E: 102.936338°	3	12
25	Middle of Teniza	3727.4	N: 34.000055°	E: 102.933488°	10	45
26	Top of Teniza	3797.9	N: 33.994798°	E: 102.936672°	9	66
27	Teniza ranch	3690.2	N: 33.989140°	E: 102.923732°	1	2
28	Xiawuka	3544.9	N: 34.276282	E: 103.002623	15	68
29	Zagana village	2939.3	N: 34.234969°	E: 103.194133°	12	40
30	Zagana moutain	3492.3	N: 34.245820°	E: 103.136100°	10	20
31	Top of Zirun valley	3620.5	N: 34.154483°	E: 103.348363°	10	71
32	Zirun valley	3512.2	N: 34.155564°	E: 103.353332°	8	28
33	Zirun ranch	3409.6	N: 34.148988°	E: 103.352220°	8	23
34	Middle of Zirun valley	2984.7	N: 34.083045°	E: 103.307977°	8	18
Total					27	1106
